# Inverse design of core-shell particles with discrete material classes using neural networks

**DOI:** 10.1038/s41598-022-21802-3

**Published:** 2022-11-08

**Authors:** Lina Kuhn, Taavi Repän, Carsten Rockstuhl

**Affiliations:** 1 Institute of Theoretical Solid State Physics, Karlsruhe Institute of Technology, 76131 Karlsruhe, Germany; 2 Institute of Physics, University of Tartu, W. Ostwald St 1, 50411 Tartu, Estonia; 3 Institute of Nanotechnology, Karlsruhe Institute of Technology, 76344 Eggenstein-Leopoldshafen, Germany

**Keywords:** Nanophotonics and plasmonics, Metamaterials, Micro-optics, Sub-wavelength optics

## Abstract

The design of scatterers on demand is a challenging task that requires the investigation and development of novel and flexible approaches. In this paper, we propose a machine learning-assisted optimization framework to design multi-layered core-shell particles that provide a scattering response on demand. Artificial neural networks can learn to predict the scattering spectrum of core-shell particles with high accuracy and can act as fully differentiable surrogate models for a gradient-based design approach. To enable the fabrication of the particles, we consider existing materials and introduce a novel two-step optimization to treat continuous geometric parameters and discrete feasible materials simultaneously. Moreover, we overcome the non-uniqueness of the problem and expand the design space to particles of varying numbers of shells, i.e., different number of optimization parameters, with a classification network. Our method is 1–2 orders of magnitudes faster than conventional approaches in both forward prediction and inverse design and is potentially scalable to even larger and more complex scatterers.

## Introduction

The interaction of light with small particles in the wavelength to sub-wavelength regime is a powerful tool to manipulate and shape light purposefully^1–3^. Hence, there is considerable interest in the design of such particles. Whereas the forward problem, i.e., calculating the response of a given particle, can generally be treated by analytical or numerical means^4^, the inverse design remains a major challenge^5–7^. Common approaches often demand repeatedly time-consuming and computationally costly calculations, thus the development of alternative methods is crucial. Recently, machine learning achieved major breakthroughs in several scientific fields, including nano-photonics^8–13^. This covers but is not limited to the design of nano-photonic devices like metasurfaces and materials^14–17^, nano-particles^18,19^ or optical cloaks^20^. Additionally, machine learning based methods can act as forward predictors of high accuracy, yielding significantly lower runtimes than conventional methods^21–24^. In this paper, we present a data-driven machine learning-based approach to predict the scattering response as well as the inverse design of spherical particles with a scattering response on demand. Nanoparticles with distinct scattering behavior and Mie resonances offer a powerful way to purposefully shape light and have been used for color generation^25–27^, the design of dielectric metamaterials^28,29^, nanoantennas^30^, or radiative daytime cooling^31^.

The interaction of light with spherical particles can be solved analytically starting from Maxwell’s equations and has been derived first by Gustav Mie in 1908^32,33^. Nevertheless, the calculations can be time-consuming, whereas artificial neural networks (ANNs) have proven to have the potential to speed up respective computations tremendously^18,19,24^. Moreover, given a reasonable amount of training data, ANNs can learn and adapt to functions as fast forward predictors. However, the inverse problem poses a more challenging issue. Not only the existence of a particle for the desired response is uncertain, but also several solutions can be possible. Especially the non-uniqueness of the problem aggravates the training of direct inverse ANNs and demands special treatments or architectures, for example, a tandem configuration^34^. Additionally, the design of scatterers involves optimizing both continuous geometrical parameters and discrete materials to achieve experimentally feasible designs.

In this work, we propose a machine learning-assisted data-driven approach for designing core-shell particles within a restricted geometrical range and considering fixed material classes. Specifically we introduce a novel two-step optimization scheme to handle continuous (shell thickness) and discrete parameters (material class) simultaneously. We successfully train ANNs to act as fast forward approximators for a given core-shell particle. Subsequently, we discuss their application as surrogate models in a global optimization algorithm. By that, we can circumvent the one-to-many problem and potentially achieve several results for a given target, an issue frequently appearing in inverse problems^4,21,35^.We benchmark our results to a conventional approach, i.e., using the exact analytical computation instead of the networks. Finally, we also include the optimization of particles with various numbers of layers to expand the design space further. In the context of machine learning-based inverse design, covering several number of shells is particularly challenging. It implies a varying number of optimization parameters which usually can’t be handled by a single network where the number of input values is fixed. The designed structures can find many applications, ranging from building blocks to realize structure colors^36,37^, as light managing structures in photovoltaic applications^38–40^, super-scatterers^41,42^, or many more.

## Results

### Forward approximation

We consider the elastic scattering response of a single core-shell particle consisting of a core and four shells. The thickness of each layer is constrained. Hence, the core radius ranges from 50 nm to 100 nm and the shell thickness from 30 nm to 50 nm. We include five different lossless and non-dispersive materials labeled from one to five in increasing order concerning the refractive index, see table 1. The resulting scattering response spectrum is discretized at 200 points within 400–800 nm wavelength. In the case of spherical scatterers, an analytical solution for the described particles can be deduced using Mie theory^43^.

**Figure 1:**
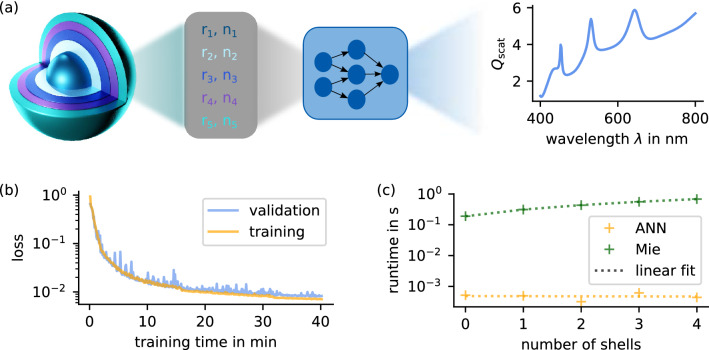
(**a**) Forward approximation of elastic scattering response of a core-multi-shell-particle with an artificial neural network. The particle is parametrized in five geometrical and five material parameters that comprise the network’s input. Subsequently, the network predicts the scattering efficiency at 200 wavelengths within a spectral interval between 400 nm and 800 nm. (**b**) Training and validation loss with a final MSE of $8.24\times 10^{-3}$. (**c**) Runtime comparison of analytical calculation using Mie-theory and ANN approximation.

First, we train a neural network to predict the scattering response of a core-shell particle in the described parameter range, see Fig. 1a. We set up a fully connected feed-forward network with five hidden layers and a total of $\sim 175,000$ trainable parameters. The input consists of five geometrical parameters, the thickness of each layer, and five material parameters, the refractive index classes. The output comprises the scattering efficiency $Q_{\mathrm {scat}}(\lambda )$ at 200 discrete points within the given wavelength range. We generate 50, 000 samples for training and 10, 000 samples each for validation and testing, where one sample comprises 200 wavelengths. To find suitable hyperparameters, we use the optuna framework^44^. The training progress is depicted in Fig. 1b, showing a convergence of both training and validation loss within 40 minutes to a final mean squared error (MSE) of $\frac{1}{N}\sum _i^N (\hat{Y}_i-Y_i)^2 = 8.24\times {10}^{-3}$. Here, $Y_i$ is the correct and $\hat{Y}_i$ the approximated spectrum of the network in the validation set labeled with index *i*. To probe the accuracy of our model, we additionally calculate the mean relative error (MRE) using 10, 000 test samples defined as $\frac{1}{N} \sum _i^N \left| (Y_i-\hat{Y}_i)/Y_i \right|$. This results in a mean deviation of 2.1%, proving that the network can approximate the scattering response with high precision.

Although there exists an exact analytical solution, the use of neural networks as forward approximators exhibits advantages regarding the processing time. We train several networks on different particle shell numbers and compare the runtime to analytical computations. Figure 1c shows the mean computing time using 1, 000 samples for each particle configuration, performed on a *Intel Core i7-7700-CPU*. The processing time with Mie calculation scales linearly with increasing shell number and is two orders of magnitude slower compared to the ANNs. Similar observations and runtime reductions for forward approximation of light-matter interactions have been made in related works^19,23,45^. Furthermore, due to the commensurable number of trainable parameters in every network, their computing time is comparable and independent of the particle configuration. Since the analytical computation for a single configuration is still less than a second the further reduction by neural networks appears to be not too advantageous at a first glance. However, when it comes to the design of scatterers, conventional methods often rely on many consecutive function evaluations. Here, a reduction of the runtime by two orders of magnitude is absolutely significant. Of course, one has to regard the amount of time needed for data generation and training. Fortunately, the training samples can be generated in parallel and although the training takes 40 min, once trained, the models are much faster and can be utilized for many optimizations, thus they can save a significant amount of time in total. A demonstrative comparison of our optimization method using networks versus analytical code is discussed in a later section.

**Table 1:** Different materials, associated classes and refractive indices.

Class	1	2	3	4	5
Material	SiO$_2$	MgO	ZnO	ZrO$_2$	TiO$_2$
Refractive index	1.465	1.720	1.945	2.074	2.431

**Table 2:** Optimized particle parameters targeting an example configuration after the first and second optimization step.

	Thickness in nm					Material class					MRE$_\mathrm {opt}$
Target	95	34	42	43	49	5	1	2	3	4	–
First step	95.36	37.10	42.62	41.38	44.64	4	3	1	4	4	1.96%
Second step	100.00	35.78	41.59	40.92	42.70	4	3	1	4	4	1.07%

**Figure 2:**
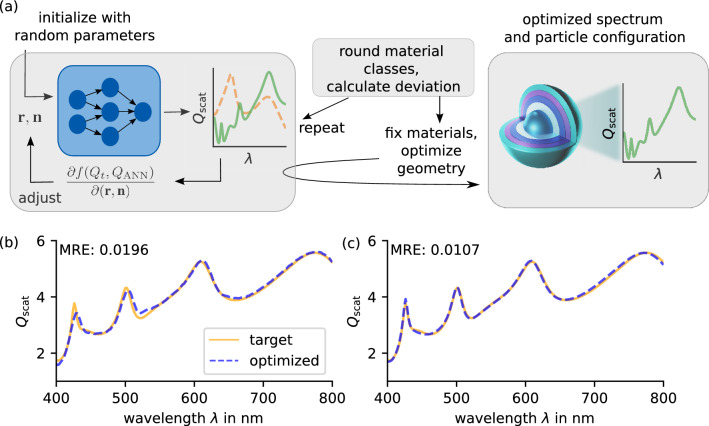
(**a**) Inverse design optimization scheme targeting the scattering response of a core-shell particle made from a core and four shells. In the first step, we initialize the neural network with some random parameters and minimize the deviation of the resulting output spectrum and the target by varying all input parameters. If the final spectrum is close enough to the target, we round and fix the material classes and fine-tune the geometry in a second optimization step. Example optimization and its results after the first (**a**) and second (**b**) optimization steps. The parameters used in this example are shown in Table 2.

### Inverse design

We use our trained models to inversely design a core-shell particle targeting a dedicated scattering efficiency. Therefore, the neural network functions as a surrogate model embedded in the gradient-based limited-memory Broyden-Fletcher-Goldfarb-Shanno algorithm including boundary constraints (L-BFGS-B)^46–50^. A schematic depiction is shown in Fig. 2a. We initialize the search with some random particle parameters and compute the resulting spectrum using the trained model. Subsequently, we compare target and output spectrum by calculating their mean absolute error (MAE), which serves as the objective function *f*

1 $$\begin{aligned} f(Q_{\mathrm {target}}, Q_{\mathrm {ANN}};{\textbf {r}},{\textbf {n}}) & = \mathrm {MAE}(Q_{\mathrm {target}}, Q_{\mathrm {ANN}};{\textbf {r}},{\textbf {n}})\,. \end{aligned}$$

To minimize the objective, we need the gradients of *f* with respect to the input parameters ${\textbf {r}}$ and ${\textbf {n}}$. Since every operation within the network is differentiable, we compute the gradients using PyTorch’s automatic differentiation package autograd. According to the gradients, the input parameters get adjusted.

However, this approach exhibits some problems that require special treatment. To handle the simultaneous optimization of discrete material and continuous geometrical parameters, we divide the optimization into two steps. In the first step, we do not restrict the material parameters to integers but allow float values. Here, we assume a well-behaved interpolation of the trained networks between spectra of different material classes, which is tested and confirmed on several examples. Given a smooth transition of the spectra, we assume a similar behavior for the optimization landscape, thus a continuous treatment of the classes doesn’t aggravate the design process. Subsequently, we round the resulting optimized material parameters to the closest integer values and fine-tune the geometry parameters in a second optimization step. Besides, we observe a non-negligible sensitivity to the initialization parameters that strongly affects the quality of the optimization result. Fortunately, a single optimization usually takes less than a second, thus we can perform several first optimization steps with different initialization parameters until we obtain a reasonable result. However, we have to limit the number of initial trials to avoid long computing times in the case of unfortunate or inaccessible targets.

Figure 2b,c shows an example optimization targeting the scattering efficiency of a known core-shell particle, see table 2. The first optimization step needs eight initializations until the success criterion is fulfilled, shown in Fig. 2a. After rounding the material classes, we achieve a deviation of 1.96%. The fine-tuning of the geometrical parameters reduces the error by a factor of $\sim 2$ to a final value of 1.07%, depicted in Fig. 2b. The entire optimization process takes only 1.44 s. Additionally, the comparison of optimized parameters and the target configuration emphasizes the non-uniqueness of the problem. Despite the low deviation of 1.07% of target and optimized spectrum, the particle configurations differ significantly concerning material and geometric parameters.

**Figure 3:**
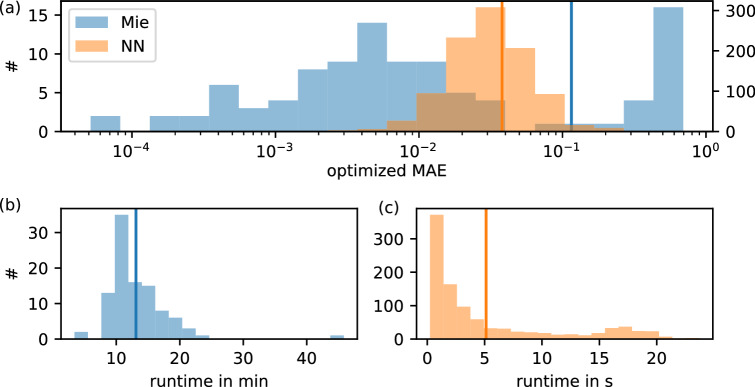
Large-scale testing using either trained models (orange) or analytical Mie calculations (blue) for inverse design. (**a**) MAE of target and optimized spectra with a mean value of $0.0379\times 10^{-2}$ using neural networks and $0.115\times 10^{-1}$ using Mie-code. (**b**, **c**) Computing time of an optimization with an average of $5.11\,$s in case of neural networks and $13.10\,$min for Mie calculations.

We test our inverse design approach using 1, 000 targets sampled from random core-shell particles within the training parameter range. By that, we can assure the existence of a solution and probe if our optimization scheme can find it. Figure 3 (a) depicts the MAE of optimized spectrum and target (orange) with a mean value of $0.0379\times 10^{-2}$. Only in a few cases, the optimized MAE is in the order of $1\times 10^{-1}$ which still can be considered close to the target. This large-scale testing shows that a maximum number of 40 initial trials is sufficient to find a design of high accuracy. The computing time for each optimization is shown in Fig. 3b with an average of $5.13\,$s.

We compare these results to the use of Mie calculations instead of ANNs in our optimization approach, see Fig. 2a. Since analytical calculations are not restricted to certain materials, we allow any value of the refractive index between material one and five. This enables us to skip the second optimization step. Unfortunately, we have to rely on numerical gradient approximation, which increases the number of function evaluations, hence the computing time. As already shown, analytical calculations take approximately 100 times longer than the trained networks, leading to computing times in the order of minutes for a single initialization. To limit the runtime reasonably, we decide to initialize the optimization only once with a single set of random particle parameters and, additionally, test it using only 100 samples. The results are shown in Fig. 3a–c. The distribution of the optimized MAE differs strongly compared to the neural network. Although the minimum values reach deviations down to $\sim 1\times 10^{-4}$, there are also cases with large errors close to one. This corresponds to a mean value of $1.15\times 10^{-1}$ and variance of $4.6\times 10^{-2}$. We assume that this behavior is caused by the single initialization, which leads to poor inverse design results in the case of unfortunate starting parameters. This problem may be solved by allowing several trials at the expense of computing time. However, the mean runtime using a single initialization is $13.1\,$minutes, which shows that additional trials are very costly, see Fig. 3b. In contrast, an optimization with ANNs takes only 5.11 s in average, including several initializations with an upper limit of 40, shown in Fig. 3c.

Comparing the overall performance of both approaches, the use of neural networks appears to be advantageous in many aspects. Although analytical calculations obtain particle configurations with lower MAE on the target, they also fail completely in some cases. Neural networks achieve more consistent inverse design results requiring significantly less computing time. For a single optimization, the proposed workflow may not be reasonable given the amount of time needed for data generation and training. However, when considering several optimizations, it pays off quickly and allows for finding several suitable designs. Additionally, the generation of the training data can be fully parallelized.

**Figure 4:**
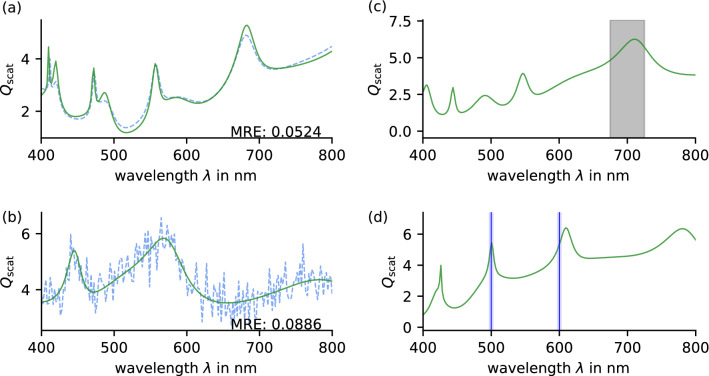
(**a**) Optimization on target spectrum (solid line) of a seven-layered core-shell particle with a deviation of 5.2%. (**b**) Optimization on noised target. (**c**) Modified objective to enhance scattering efficiency for wavelengths within 675 nm and 724 nm. (**d**) Targeting peaks at 500 nm and 600 nm.

So far, we only tested our inverse design optimization on accessible target spectra within the trained parameter range. Of course, testing the optimization on any general spectrum is likely to fail since we follow a data-driven approach that is restricted to the training data limits. However, we can probe the performance on spectra generated with core-shell particles outside the trained parameter range. This ensures target spectra similar to but not covered by the training data. For example, Fig. 4a shows an optimization on a target spectrum generated using a seven-layered core-shell particle. With a mean deviation of $\sim 5$%, the optimization result is in very good agreement with the target.

### Expansions and Modifications of the Design Approach

We want to expand our optimization procedure to core-shell particles of different numbers of layers. Unfortunately, it is challenging to realize this with a single network since the number of input parameters varies with the amount of layers. To cover various shell numbers, we decide to train several networks on particles with zero, one, ..., four shells. To determine which number of shells works best for a specific target, we additionally train a classifier that takes the desired spectrum as input and returns the best possible number of shells for the inverse design. Due to the non-uniqueness of scattering problems, the generation of the training data for the classifier is challenging. We generate 20,000 spectra of particles comprising zero up to four shells. Of course, the number of shells used for the generation of a spectrum is known. Nevertheless, a different particle configuration may result in a very similar or even identical spectrum. To regard all possible configurations, we use our presented optimization procedure. We perform several inverse designs of every generated spectrum, each with a different network, i.e., the number of shells. Finally, we compare the optimization results $S_i$ to the target *T* and use the respective MAE to define a probability for every number of shells

2 $$\begin{aligned} P(S_i) & = \left\{ \begin{array}{ll} 1 - \mathrm {MAE}(S_i, T), &\mathrm {MAE}(S_i, T) < 1 \\ 0, &\mathrm {MAE}(S_i, T) \ge 1 \end{array} \right. \,. \end{aligned}$$

Subsequently, we successfully train a classifier to approximate the probabilities for a particular target. The optimization scheme shown in Fig. 2a can now be expanded by the classifier to decide which number of shells works best, i.e., which forward approximation network to use. We test this approach on 1,000 random targets of different shell configurations and achieve an MSE of $2.19\times 10^{-2}$. We manually examine the worst optimizations to check whether a wrong classification causes the results. It appears that this is not the case, and the correct number of shells is suggested, concluding that an unfortunate choice of the initial or target spectrum caused the poor results.

We now want to test the generalizability and robustness of our approach using spectra created with parameters outside the training range and noisy targets. Figure 4a shows the result using a core-shell particle restricted to the trained materials but comprising seven layers. Nevertheless, the optimization provides a 5-layered design with a deviation of only 5.24%. Additional tests reveal that our approach can find a decent design for targets created with more than five layers providing that the total radius of the particle is not larger than the maximum radius of the training range.

To test the robustness against noise, we add a Gaussian error $N(\mu = 0, \sigma = 0.5)$ to every point of the spectrum and perform the design. Figure 4b shows an example using four layers. The deviation of 8.86% refers to the noisy spectrum and the design. The comparison to the unperturbed spectrum yields an even lower relative error of 1.27%. Especially the performance of the classifier with noisy spectra is remarkable since we never used them in training.

In general, we can target any spectrum within the defined wavelength regime. However, the resulting design is limited to feasible spectra provided by the particles the networks are trained on. If the desired spectrum is not accessible in our design space, the optimization may fail or return a particle with a similar response but not exact response. Fortunately, in many cases it is sufficient to match only for some wavelengths or maximize or minimize the scattering efficiency in a certain range which we will discuss in the next sections.

One of the approach’s advantages is the versatile objective function that can be modified according to specific needs. By that, it is possible to target not only a certain spectrum but rather an area of enhancement or even isolated resonance wavelengths

3 $$\begin{aligned} f(\lambda _{\mathrm {in}}) & = \frac{\overline{Q_{\mathrm {scat}}(\lambda _{\mathrm {out}})}}{\overline{Q_{\mathrm {scat}}(\lambda _{\mathrm {in}})^2}}\,. \end{aligned}$$

Here, $\lambda _{\mathrm {in}}$ represents either a single or several target wavelengths and respective widths. Figure 4c,d show two example optimizations using a modified objective. In (c) we optimize on a broad enhancement region of wavelength $\lambda _\mathrm {in} = \left( 700\pm 25\right) \,$nm, whereas in (d) we search for two sharper resonances of line width $5\,$nm at $\lambda _1 = 500\,$nm and $\lambda _2 = 600\,$nm. Considering all multipolar orders, the designs provide only a limited attenuation of the scattering efficiency outside the enhanced region restricted to accessible spectra within the training range. The same applies when addressing several resonance wavelengths. Whereas a single resonance usually can be found, certain combinations may not be feasible.

## Discussion and conclusion

We successfully trained neural networks to predict the scattering response of core-shell particles made from several discrete material classes within a certain geometry range. The approximation yields high accuracy and, moreover, requires only a fraction of the processing time compared to analytical calculations. Subsequently, we have proposed a new inverse design approach that covers the optimization of both continuous and discrete particle parameters. We have used the networks as surrogate models in a global gradient-based minimization algorithm, and we have taken advantage of the fast and analytical gradient extraction given the differentiability of the ANNs. In a two-step procedure, we first continuously optimize all parameters exploiting the smooth interpolation of the ANNs between two material classes. Following this, we perform a rounding and fixing of the materials and complete the design with a second optimization, i.e., fine-tuning, of the geometric parameters. Finally, we handle the sensitivity to the choice of the initial parameters by enabling several trials until a success criterion is met. Large-scale testings proved that our approach consistently provided designs with low errors on given target spectra, taking only a few seconds on average. 0f course, one has to regard the time needed for data generation and training, but given the runtime, the optimization with analytical code takes, the point of amortization is already reached within a few designs.

Finally, we expanded our method to particles of different layer counts and trained an additional classifier to suggest a suitable number of layers given a target spectrum. We use our developed optimization approach to generate the respective training data to treat the non-uniqueness of the problem. By that, we further expanded the design space handled the problem of a varying number of optimization parameters itself. Furthermore, we can modify the objective function according to needs which allows us to search for broad scattering enhancement regions or isolated resonances. Finally, we test our approach on targets outside the trained parameter range as well as noisy spectra and still achieve very good results. Due to the versatility of our proposed method, it is possible to expand the training data to even larger particles and further materials.

## Data Availability

The data, trained networks and code that support this study will be made available on GitLab upon publication https://github.com/tfp-photonics/cs_optim. It is also available on reasoable request from the corresponding author.
